# RTCGAToolbox: A New Tool for Exporting TCGA Firehose Data

**DOI:** 10.1371/journal.pone.0106397

**Published:** 2014-09-02

**Authors:** Mehmet Kemal Samur

**Affiliations:** 1 Department of Biostatistics and Computational Biology, Dana-Farber Cancer Institute and Harvard School of Public Health, Boston, Massachusetts, United States of America; 2 Lebow Institute of Myeloma Therapeutics and Jerome Lipper Multiple Myeloma Center, Dana-Farber Cancer Institute and Harvard Medical School, Boston, Massachusetts, United States of America; Huazhong University of Science and Technology, China

## Abstract

**Background & Objective:**

Managing data from large-scale projects (such as The Cancer Genome Atlas (TCGA)) for further analysis is an important and time consuming step for research projects. Several efforts, such as the Firehose project, make TCGA pre-processed data publicly available via web services and data portals, but this information must be managed, downloaded and prepared for subsequent steps. We have developed an open source and extensible R based data client for pre-processed data from the Firehouse, and demonstrate its use with sample case studies. Results show that our RTCGAToolbox can facilitate data management for researchers interested in working with TCGA data. The RTCGAToolbox can also be integrated with other analysis pipelines for further data processing.

**Availability and implementation:**

The RTCGAToolbox is open-source and licensed under the GNU General Public License Version 2.0. All documentation and source code for RTCGAToolbox is freely available at http://mksamur.github.io/RTCGAToolbox/ for Linux and Mac OS X operating systems.

## Introduction

The explosion of data from high throughput experiments, fueled by various functional genomics technologies, is expected to overwhelm attempts at analyzing genomics data [1], [2]; this trend is most evident in oncogenomics, where a vast number of tumors have been profiled by individual laboratories. By the end of 2015, the Cancer Genome Atlas (TCGA) (http://cancergenome.nih.gov) [3] Research Network plans to achieve the ambitious goal of analyzing the genomic, epigenomic and gene expression profiles of more than 10,000 specimens from more than 25 different tumor types [4]. The massive amounts of information that is emerging from such large-scale project is becoming increasingly difficult for researchers to manage.

In 2013, TCGA Research Network summarized the aims of TCGA project as to generate, quality control, merge, analyze and interpret molecular profiles at the DNA, RNA, protein and epigenetic levels for hundreds of clinical tumors representing various tumor types and their subtypes [4]; the authors also reported that cases that meet quality assurance specifications are characterized using technologies that assess the sequence of the exome, copy number variation, DNA methylation, mRNA expression and sequence, microRNA expression and transcript splice variation. Additional platforms applied to a subset of the tumors, including whole-genome sequencing and RPPAs, provide additional layers of data to complement the core genomic data sets and clinical data [4].

Such a deluge of data also creates problem of access and management for researchers. A key factor in the utility, sustainability and future use of a novel resource lies in its ability to allow for data sharing and to be interoperable with major international cancer research efforts [5]. In addition, Buetow et. al. and Saltz et. al. also underscore the importance of interoperable IT infrastructures that facilitate simpler data access and data sharing for cancer research [6], [7]. To address these challenges, a number of tools for different genomic data platforms have been developed by several groups: these include GEOquery [8], BioMart (a simple federated query system based on a generic framework designed for biological storage and retrieval) [9], [10] and web based tools such as an engine to index and annotate the TCGA files [11].

A limited number of web portals (such as canEvolve [2] and cBio [12], [13]) are available to access and organize TCGA data for further analysis. The Firehose pipeline management system has been developed by the Broad Institute (http://gdac.broadinstitute.org), for use in comprehensive automated and reproducible analyses of the data generated by TCGA [14]. However, even though Firehose provides pre-processed data to the research community, it has several limitations with regards to systematic access to the data, and many researchers write their own (or borrow) shell, Perl or Python scripts to download required files to their local environment [15]. Although Firehose projects provides the “firehose_get” tool, which is efficient than downloading data from web directly for pipelines and analysis tools, it is not easily integrated with programming environments for post analysis.

Here we present an open source library for access and management of TCGA data. RTCGAToolbox allows users to systematically access Firehose pre-processed data, and to organize it for easy management and analysis. Currently, Firehose allows access to more than 7 primary data types for more than 25 cancer subtypes (Table 1). The library also allows users to create data matrices from TCGA data, without any pre-processing. RTCGAToolbox can also access the Firehose analysis pipeline to get GISTIC2 [16] results for questions related to copy number data. In addition, basic analysis functions of RTCGAToolbox facilitate basic comparisons and analyses as well as visualization without having to call external tools. Furthermore, users can hire their favorite R packages to develop their own pipelines for downstream analysis with analysis-ready matrices. Several recent publications [17], [18], [19] show that systematic access and analysis of TCGA data provides valuable information about cancer and helps researchers to improve their studies.

**Table 1:** Current Firehose data content (Some of these data may not be accessible due to TCGA data restrictions, full data table can be accessible via http://gdac.broadinstitute.org/runs/stddata__2014_03_16/ingested_data.html).

Cohort	Clinical	CN	Methylation	mRNA	mRNASeq	miR	miRSeq	RPPA	MAF
ACC	15	90	80	0	79	0	80	0	90
BLCA	198	252	242	0	241	0	241	127	130
BRCA	981	1041	1024	526	1037	0	1021	408	976
CESC	127	192	189	0	185	0	200	0	39
COAD	436	427	434	153	432	0	406	331	154
COADREAD	604	589	596	222	595	0	549	461	223
DLBC	21	28	28	0	28	0	27	0	0
ESCA	39	97	93	0	0	0	72	0	0
GBM	578	570	414	540	160	565	0	214	290
HNSC	408	509	457	0	497	0	512	212	306
KICH	93	66	66	0	66	0	66	0	66
KIRC	507	514	511	72	518	0	502	454	417
KIRP	164	182	198	16	172	0	198	0	168
LAML	200	197	194	0	179	0	188	0	197
LGG	305	463	403	27	463	0	438	258	289
LIHC	151	190	194	0	191	0	200	0	0
LUAD	466	493	555	32	488	0	491	237	229
LUSC	411	490	492	154	489	0	467	195	178
MESO	13	37	37	0	0	0	0	0	0
OV	580	576	584	574	296	570	453	412	316
PAAD	73	91	91	0	85	0	85	0	91
PCPG	10	0	179	0	0	0	0	0	0
PRAD	199	331	336	0	297	0	326	164	261
READ	168	162	162	69	163	0	143	130	69
SARC	102	137	170	0	103	0	136	0	0
SKCM	341	385	374	0	372	0	354	205	344
STAD	311	352	373	0	274	0	323	264	221
THCA	484	494	496	0	494	0	495	222	402
UCEC	482	525	532	54	527	0	513	200	248
UCS	57	56	57	0	57	0	56	0	57
Totals	7920	8947	8965	2217	7893	1135	7993	4033	5538

## Implementation

**Figure 1:**
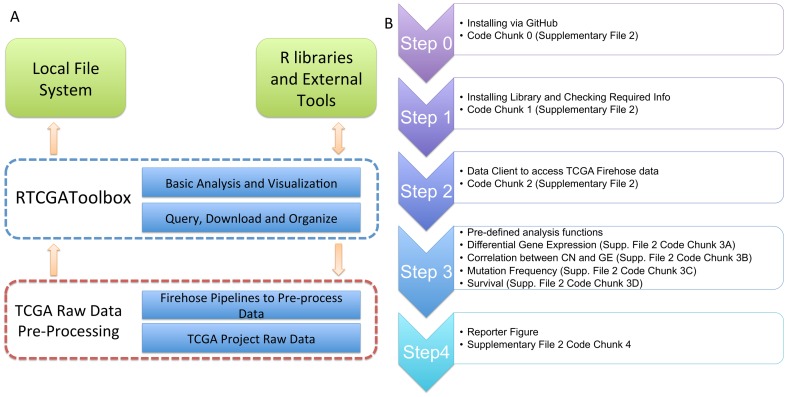
Overall RTCGAToolbox structure and workflow. (A) Overall representation of RTCGAToolbox layers from Firehose web portal to user environments. (B) Sample workflow for “BRCA” dataset.

Development of the RTCGAToolbox package was mainly driven by two major demands: (i) to provide a user-friendly and rapid data access to TCGA data processed by Firehose; and (ii) to provide a programmatic interface for analysis software/pipelines to access TCGA data systematically. RTCGAToolbox is developed by using R programming language and provides expandable open source environment for future development and integration. Figure 1A shows a schematic overview of the RTCGAToolbox and its basic functionalities.

RTCGAToolbox uses the Firehose project, one of the largest TCGA data sources operated by Broad Institute's Genome Data Analysis Center (GDAC), to access Level 3 (segmented or interpreted data) and Level 4 (region of interest data) pre-processed data.

The first level of processing is to access Firehose reports, and prepare datasets and type lists in order to organize data. The main module of the RTCGAToolbox accesses reports via HTTP calls, and uses text processing functionalities to prepare required information for subsequent steps. To support further analysis, the RTCGAToolbox creates data object by parsing the default Firehose exports; this function can be useful for possible future integrations with other environments and R resources. Client function also allows users to access different Firehose archive dates programmatically. In addition to these data client functionalities, the current version of RTCGAToolbox facilitates basic analysis (differential gene expression, mutation frequencies, survival analysis and copy number and gene expression correlation analysis). Also with the S4 class defined output objects users can use their favorite algorithm and packages to undertake further downstream analysis.

## Description

To use the RTCGAToolbox as a data client for the Firehose project, it is necessary to know the run dates for Firehose standard data and analyses. The RTCGAToolbox provides functions to list the “standard data run”, the “analysis run”, and names for valid dataset aliases. Users must provide valid dates and dataset aliases (File S1).

One of the primary goals of this project is to allow users to systematically access and organize TCGA Level 3 and Level 4 data outputs. Through its extensible structure, the RTCGAToolbox can be integrated with R libraries, allowing R users to also integrate their data for further analysis.

In addition to its data management functionalities, RTCGAToolbox allows users to perform basic analysis: it provides quick analysis options for deriving useful information from the data, and can also create circle plots to summarize the data. After the data-downloading step, RTCGAToolbox deletes already used compressed files, to free up disk space and users can also use stored data matrix files with different environments. Detailed case studies and user instructions are included in File S1.

### RTCGAToolbox Usage and Case Studies

The current version of RTCGAToolbox can be used as an R library. Once users get the latest version, data client and basic analysis functions to be called via the R interface. Source code and project are currently accessible through http://mksamur.github.io/RTCGAToolbox/

#### RTCGAToolbox Data Client and Analysis Functions

As a data client tool and a functional library, RTCGAToolbox provides several functions for users to control the management process: these can be described as control, client and analysis functions.

The main aim of the control functions is to provide valid date and dataset aliases to the users, and they are also used by client functions to check parameters. The Firehose project regularly provides one stddata run per month and four analyses runs per year. To access valid dates, users can call “getFirehoseRunningDates” and “getFirehoseAnalyzeDates” functions, which provide data and analysis runs date, respectively. Dataset aliases are also important for data client functionality and the “getFirehoseDatasets” function helps users to get the valid aliases for the datasets. Table 1 lists information about current dataset aliases and contents for each dataset.

The core function of the library is the client function, also referred to as the “getFirehoseData” function, which provides a data client that checks the valid dates and aliases, gets the URL for data requested by the user, downloads the data into a working directory, and prepares the data matrices for downstream analysis. Calling the function initiates three main sub-processes. At the initial step, the function accesses Firehose services to get the URLs for user specified data types, and after which client function downloads the data from the Firehose TCGA data portal. Next, the data matrix is prepared; depending on the data type, size and connection speed, this process may take a shorter of longer time. As a default, users have to specify at least 2 parameters: “dataset” and “runDate” or/and “gistic2_Date”. The current version of the RTCGAToolbox is currently capable of handling data types that are summarized in Table 2.

**Table 2:** Data types supported by RTCGAToolbox.

Data Type (Parameter)	Description	Output Object (FirehoseData)
**Clinic**	Provides clinical information for each sample. Clinical information may include stage, survival time, sex, age and more.	fd@Clinical (data frame)
**RNAseq_Gene or/and RNAseq2_Gene_Norm**	Gene level expression data from RNA-seq platforms. This parameter provides raw counts and normalized values. Firehose provides 2 different algorithms for RNAseq data processing. (Data types can be specified by using RNAseqNorm and RNAseq2Norm parameters)	fd@ RNASeqGene, fd@ RNASeq2GeneNorm (data matrix)
**miRNASeq_Gene**	miRNA expression levels from next generation sequencing platforms	fd@ miRNASeqGene (data matrix)
**CNA_SNP**	Segmented copy number alterations (in somatic cells)	fd@ CNASNP (data frame)
**CNV_SNP**	Segmented copy number variations (in germline cells)	fd@ CNVSNP (data frame)
**CNA_Seq**	Copy number alterations provided by next generation sequencing platforms	fd@ CNAseq (data frame)
**CNA_CGH**	Copy number alterations provided by CGH array platforms	fd@ CNACGH (a list of FirehoseCGHArray objects)
**Methylation**	Methylation data provided by array platforms	fd@ Methylation (a list of FirehoseMethylationArray object)
**Mutation**	Gene level mutation information matrix	fd@ Mutations (data frame)
**mRNA_Array**	Gene level expression data provided by array platforms	fd@ mRNAArray(a list of FirehosemRNAArray objects)
**miRNA_Array**	miRNA expression data provided by array platforms	fd@ miRNAArray (a list of FirehosemRNAArray objects)
**RPPA**	Reverse phase protein array (RPPA) expression	fd@ RPPAArray(a list of FirehosemRNAArray objects)

In addition to its client functionalities, RTCGAToolbox also provides analysis functions, for collecting information from the datasets. The current version of the package comes with five basic functions: i) The “getDiffExpressedGenes” function provides the results of differential gene expression analysis. It takes sample barcodes to differentiate between “Normal” and “Tumor” samples, and compares them with linear models and empirical Bayesian methods provided by the limma [20] package. It also uses voom [21] function (from the same package) to prepare raw RNAseq counts for differential gene expression analysis. ii)Previous studies show that copy number alterations may affect the levels of gene expression[22]. Based on the dosage effect hypothesis, we have integrated the “getCNGECorrelation” function for calculateing correlations between copy number estimates from GISTIC2 [16] and gene expression levels. iii) All cancers are known to be caused by somatic mutations, however, our understanding of the mutational processes that cause these mutations is remarkably limited [23]. The “getMutationRate” function was developed to return gene mutation frequencies from samples that have mutation information. This function is useful if users want to integrate information about mutation with other data types. iv) Survival analysis is considered to be one of the methods that yields clinically valuable information. To allow the user to gain into survival profiles, the RTCGAToolbox has the “getSurvival” function, which creates sample groups by using levels of gene expression, compares differences between groups, and provides KM plots as a final product. v) And finally, the RTCGAToolbox also includes the “getReport” function, which is a visualization tool that uses differential gene expression analysis, copy number data and mutation rate to visualize genome wide alterations with RCircos [24].

#### RTCGAToolbox Case Study

We provide below a case study to show the current functions of RTCGAToolbox, and to demostrate how to integrate its outputs with other R libraries. We also provide a user guide and step by step sample code in Figure 1B, File S1 and File S2. For this case study, we analyze breast invasive carcinoma [BRCA] mRNA, copy number, mutation and clinical data with the RTCGAToolbox.

**Figure 2:**
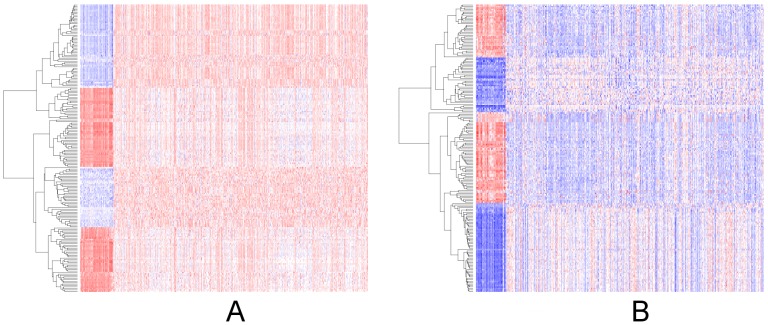
Sample heatmap outputs from BRCA dataset. Panel A and B show the top differentially up and down regulated genes between “Cancer” and “Normal” samples by using RNASeq and microarray data respectively.

**Figure 3:**
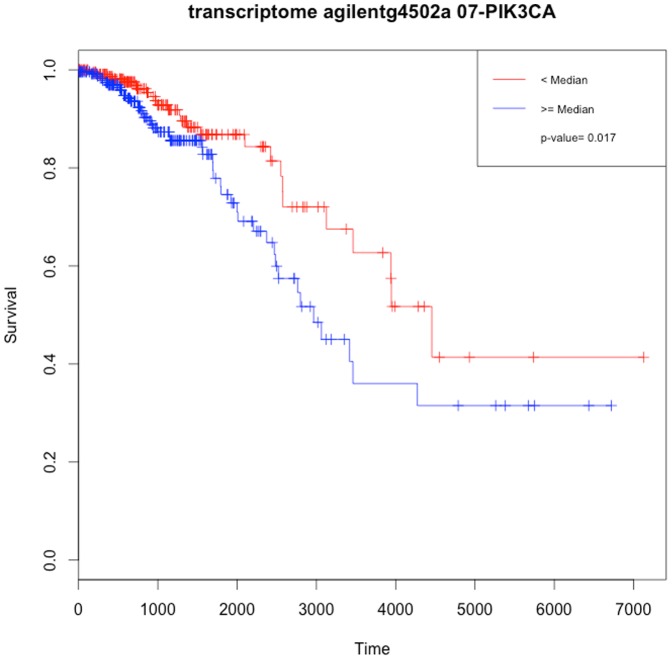
KM plot for PIK3CA gene. A KM plot that compares the survival difference between PIK3CA, which is the gene has highest mutation frequency in BRCA dataset, high and low expressed samples.

i) After installing (Figure 1B, Step 0) the library via http://mksamur.github.io/RTCGAToolbox/, the RTCGAToolbox can be called (Figure 1B, Step 1). Note that the library depends on several other R packages and these libraries must be working properly (see “Known issues” below).

ii) Users then use one valid dataset alias and stddata or/and analysis date, to call the data client. Information about additional data types and structure, with valid parameter names, is listed in Table 2.

iii) “getFirehoseData” (Figure 1B, Step 2) is the main data client function to process and prepare analysis matrices. This function returns an object that stores the requested data in matrices, lists, or data frames. After successfully requesting and getting data, analysis functions can be used to get quick results.

iv) The “getDiffExpressedGenes” (Figure 1B,Step 3A) function accepts an object produced by the “*getFirehoseData*” function. TCGA project produces systematic barcodes for each sample and the “getDiffExpressedGenes” function uses the same systematic approach to create “Tumor” and “Normal” sample groups. Users do not need to define groups separately to perform the analysis. TCGA project collects data from multiple platforms such as RNAseq and microarray platforms. If the dataset has multiple mRNA expression data from different platforms, the “getDiffExpressedGenes” function calculates the differential gene expression between the groups for each dataset separately, and returns a list that stores the results for each platform. Figure 2 also shows the heatmap outputs of differentially expressed genes. The “getDiffExpressedGenes” function also provides volcano plots. And following the analysis, the function also has the capability to filter results by using fold change and p values (Figure 1B, Step 3A), to yield strong differences between groups. Criello et al. recently showed that copy number changes are dominant in several cancer types [25]. To enable analysis of copy number variations, and to calculate the correlation between the copy number and the gene's expression level in paired samples, we added the “getCNGECorrelation” function (Figure 1B, Step 3B). The function returns a list object that stores the resulting data frames constructed by gene symbol, correlation coefficient and adjusted p values for each gene. Criello et al. also point out that mutations also dominate several cancer types [25]; however, our understanding of the mutational processes that cause somatic mutations in most cancer classes is remarkably limited [23]. We thus incorporated the “getMutationRate” function for calculating the frequency of gene mutations (Figure 1B, Step 3C). And finally, the RTCGAToolbox uses univariate survival analysis (Figure 1B, Step 3D) and KM plots to show differences in survival associated with high and low levels of expression of individual genes. To run the survival function, users must provide a data frame that includes a sample barcode, time, and event data. This frame can be obtained from clinical data, which can be downloaded by use of the data client function. Figure 3 shows the KM plot from the output of the survival function.

**Figure 4:**
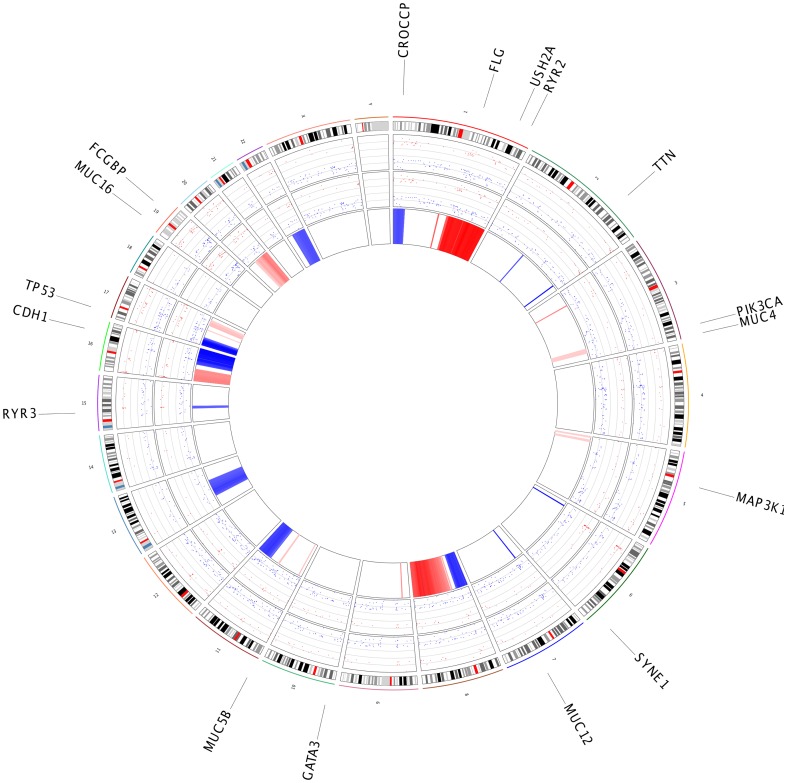
Summary plot for BRCA dataset. A circle plot that shows the differentially expressed genes result from RNASeq and microarray platform (Inner circle 1 and 2, y axis represents the fold change value, red dots are up regulated and blue dots are down regulated in cancer samples), copy number changes (inner third circle, blue zones represents the deletions and red circle represents the amplifications) and outer circle shows the genes that has mutation at least 5% of samples.

v) To provide a visual summary for each dataset, we have implemented a reporter function that creates a circle plot, developed for large-scale multi-sample genomic research data [24]. The “getReport” function (Figure 1B, Step 4) uses data about the copy number, mutations, and results from differential gene expression analysis results to produce Figure 4, the summary figure for the BRCA dataset. The outer circle shows the gene symbols that are mutated in at least 5% of the samples; inner track shows the significant alterations in gene expression, as fold change and copy number changes (blue represents the deletions; red represents the amplifications).

## Known Issues and Future Work

The current version of the RTCGAToolbox was developed by using R programming language. All dependent packages must be properly working. Firehose project provides data as “TAR” archives and R core functions have been used to download and untar the files. Due to long archive file names and paths, the lient function throws numerous errors on a Windows platforms, and because of unsupported long file paths, the current version does not support a Windows environment. In addition, Mac OS Mavericks may use a different TAR path, in which case, Mac users should also set their R configurations to [ Sys.setenv (TAR = ‘/usr/bin/tar') ].

The RTCGAToolbox currently does not support exon and isoform level data from any platforms, due to high data volume. We are planning to improve the project for these data types, in order to increase effectiveness and performance.

Prospective efforts will focus on developing integrated data analysis tools that include network analysis and visualization tools. Furthermore, we will work on implementing functions for dealing with missing data. Such integration efforts will help researchers to rapidly and easily interpret TCGA data for their own research. In addition, there are huge efforts to share the results with the community, such as cBio [12], canEvolve [2] or Oncomine [26]. Besides, RTCGAToolbox's customizable environment will also help researchers to set up their analysis pipelines by using TCGA data for their hypothesis based questions, and to enable visualization with advanced tools like Circos [27].

## Supporting Information

File S1 **RTCGAToolbox user guide.**
(PDF)

File S2 **Sample R code for re-producing the case study.**
(TXT)
