# Supplementary material for: RTCGAToolbox: A New Tool for Exporting TCGA Firehose Data
Source: PLoS One. 2014 Sep 2;9(9):e106397. doi: 10.1371/journal.pone.0106397 (PMC4152273; doi:10.1371/journal.pone.0106397)
Supplement: File S1 — RTCGAToolbox user guide. (PDF) [file pone.0106397.s001.pdf]

RTCGAToolbox Package

Mehmet Kemal Samur, Ph.D.
Department of Biostatistics and Computational Biology,
Dana Farber Cancer Institute and Harvard School of Public Health,
Boston, MA

July 24, 2014

Contents

1 Introduction **1**

2 Installation **2**
2.1 With devtools . . . 3
2.2 Manuel Installation . . . 3

3 Data Client **3**

4 Post analysis functions **4**
4.1 Differential gene expression . . . 4
4.2 Correlation between gene expression and copy number data . . . 6
4.3 Mutation frequencies . . . 6
4.4 Univariate survival analysis . . . 7
4.5 Report figure . . . 8

5 Data objects **9**

6 sessionInfo **10**

1 Introduction

Managing data from large scale projects such as The Cancer Genome Atlas (TCGA)[1] for further analysis is an important and time consuming step for research projects. Several efforts, such as Firehose project, make TCGA pre-processed data publicly available via web services and data portals but it requires managing, downloading and preparing the data for following steps. We developed an open source and extensible R based data client for Firehose Level 3 and Level 4 data and demonstrated its use with sample case studies. RTCGAToolbox could improve data management for researchers who are interested

with TCGA data. In addition, it can be integrated with other analysis pipelines for further data analysis.

RTCGAToolbox is open-source and licensed under the GNU General Public License Version 2.0. All documentation and source code for RTCGAToolbox is freely available.

Currently, following functions are provided to access datasets and processed datasets.

- Control functions:
  - getFirehoseRunningDates: This function can be called to access valid stddata run dates. To access data, users have to provide valid dates.
  - getFirehoseAnalyzeDates: This function can be called to access valid analyze run dates. To access data, users have to provide valid dates. This function only affects the GISTIC2 [2] processed copy estimate matrices.
  - getFirehoseDatasets: This function can be called to access valid dataset aliases.
- Data client function:
  - getFirehoseData: This is the core function of the package. Users can access Firehose processed data via this function. Once it is called, several steps are realized by the library to access data. Finally this function returns an S4 object that keeps all the downloaded data.
- Analysis Functions:
  - getDiffExpressedGenes: This function takes "FirehoseData" object as an input and uses differential gene expression analysis to compare cancer and normal samples. Function takes "limma"[3-4] package advantages for performing analysis. In addition, sample and normal population is obtained from TCGA sample barcodes.
  - getCNGECorrelation: This function calculates the correlation between gene expression values and copy number data. Users have to download GISTIC2 [2] copy number estimates, as well as the expression data from at least one platform.
  - getMutationRate: From all samples that have mutation information, this function calculates the genes' mutation frequency.
  - getSurvival: Performs an univariate survival comparison for individual genes between high and low expressed sample groups.

– getReport: Creates a circular pdf figure from differential gene expression, copy number and mutation information.

After successful installation of RTCGAToolbox, one needs to load the library to get started using it:

```
> library(RTCGAToolbox)
```

2 Installation

To install RTCGAToolbox, you may use either install_github function from devtools (`http://cran.r-project.org/web/packages/devtools/index.html`) package or you may download, build and install by yourself.

2.1 With devtools

```
> # You should install devtools package before loading it.
> library("devtools")
> # Next function will download, build and install RTCGAToolbox.
> install_github("mksamur/RTCGAToolbox")
> # Finally call the RTCGAToolbox
> library("RTCGAToolbox") # Package should be successfully installed!
```

2.2 Manuel Installation

First you need to download archive (zip or tar.gz) from `http://mksamur.github.io/RTCGAToolbox/`. After downloading, extract archive file into a folder. Please note that you should have all dependent packages. If you have missing dependent package, you will get an error that tells you which packages you should install. X.Y.Z is the current version of RTCGAToolbox.

Listing 1: Manuel Installing

```
cd /path/to/folder/
R CMD build RTCGAToolbox/
R CMD install RTCGAToolbox_X.Y.Z.tar.gz
```

3 Data Client

Before getting the data from Firehose pipelines, users have to check valid dataset aliases, stddata run dates and analyze run dates. To provide valid information RTCGAToolbox comes with three control functions. Users can list datasets with "getFirehoseDatasets" function. In addition, users have to provide stddata run date or/and analyze run date for client function. Valid dates are accessible via "getFirehoseRunningDates" and "getFirehoseAnalyzeDates" functions. Below code chunk shows how to list datasets and dates.

```
> library(RTCGAToolbox)
> # Valid aliases
> getFirehoseDatasets()
 [1] "ACC"      "BLCA"     "BRCA"     "CESC"     "COAD"     "COADREAD"
 [7] "DLBC"     "ESCA"     "GBM"      "HNSC"     "KICH"     "KIRC"
[13] "KIRP"     "LAML"     "LGG"      "LIHC"     "LUAD"     "LUSC"
[19] "MESO"     "OV"       "PAAD"     "PCPG"     "PRAD"     "READ"
[25] "SARC"     "SKCM"     "STAD"     "THCA"     "UCEC"     "UCS"
> # Valid stddata runs
> stddata = getFirehoseRunningDates()
> stddata
 [1] "20140614" "20140518" "20140416" "20140316" "20140215" "20140115"
 [7] "20131210" "20131114" "20131010" "20130923" "20130809" "20130715"
[13] "20130623" "20130606" "20130523" "20130508" "20130421" "20130406"
[19] "20130326" "20130309" "20130222" "20130203" "20130116" "20121221"
[25] "20121206" "20121114" "20121102" "20121024" "20121020" "20121018"
[31] "20121004" "20120913" "20120825" "20120804" "20120725" "20120707"
[37] "20120623" "20120606" "20120525" "20120515" "20120425" "20120412"
[43] "20120321" "20120306" "20120217" "20120124" "20120110" "20111230"
[49] "20111206" "20111128" "20111115" "20111026"
> # Valid analysis running dates (will return 3 recent date)
> gisticDate = getFirehoseAnalyzeDates(last=3)
> gisticDate
[1] "20140416" "20140115" "20130923"
```

When the dates and datasets are determined users can call data client function ("getFirehoseData") to access data. Current version can download multiple data types except ISOFORM and exon level data due to their huge data size. Below code chunk will download BRCA dataset with clinical, gene expression (both from RNASeq and mRNA array platfroms), mutation and GISTIC2 [2] processed copy number estimates data.

```
> # BRCA data with mRNA (Both array and RNASeq), GISTIC processed copy number data
> # mutation data and clinical data
> # (Depends on bandwidth this process may take longer time)
> brcaData = getFirehoseData (dataset="BRCA", runDate="20140416", gistic2_Date="20140115",
+                Clinic=TRUE, RNAseq_Gene=TRUE, mRNA_Array=TRUE, Mutation=TRUE)
```

4 Post analysis functions

RTCGAToolbox has analyze functions to provide basic information about datasets. Analyze function includes differential gene expression analyze, correlation analysis between CN and GE data, univariate survival analysis, mutation frequency table and report figure.

4.1 Differential gene expression

RTCGAToolbox hires voom[4] and limma[3] package functions to preform differential gene expression analysis between ”Normal” and ”Cancer” tissue samples. Every sample which is processed by TCGA project[1] has a structured barcode number which includes the source of the tissue. RTCGAToolbox uses the barcode information to divide samples into ”Normal” or ”Tumor” groups and perform DGE analysis. Since ”voom”[4] requires raw count for RNASeq data, normalized RNASeq data cannot be used for the analysis.
This function uses all gene expression datasets and returns a list which each member is ”DGEResult” object. Each result object keeps top table from the genes that have 2 log fold change expression difference and significant adjusted p value.
This function filters the results as a deafult behaviour using raw p value, adjusted p value and log fold change. Users can change ”adj.pval”, ”raw.pval” and ”logFC” parameters to refine their results. Also function uses Benjamini Hochberg adjustment for p values. For more details about adjment users can check base adjustment methods by calling ”?p.adjust”. In addition to filter as a default behaviour function only draws heatmap for top 100 up and down regulated genes. Users can also adjust these values by using ”hmTopUpN” and ”hmTopDownN” parameters.

```
> # Differential gene expression analysis for gene level RNA data.
> diffGeneExprs = getDiffExpressedGenes(dataObject=brcaData,DrawPlots=TRUE,
+                                  adj.method="BH",adj.pval=0.05,raw.pval=0.05,
+                                  logFC=2,hmTopUpN=100,hmTopDownN=100)
> # Show head for expression outputs
> for(i in length(diffGeneExprs))
+ {
+   writeLines(diffGeneExprs[[i]]@Dataset)
+   head(diffGeneExprs[[i]]@Toptable)
+ }
```

If ”DrawPlots” set as TRUE, running code above will provide following image outputs.

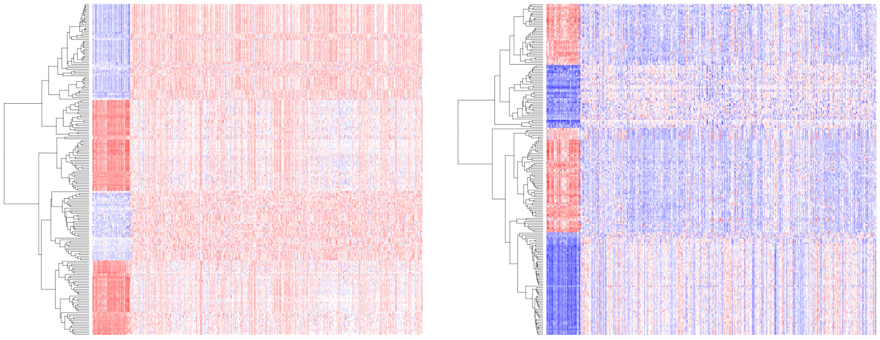

Voom + limma: To voom (variance modeling at the observational level) is to estimate the mean-variance relationship robustly and non-parametrically from the data. Voom works with log-counts that are normalized for sequence depth, in particular with log-counts per million (log-cpm). The mean-variance is fitted to the gene-wise standard deviations of the log-cpm, as a function of the average log-count. This method incorporates the mean-variance trend into a precision weight for each individual normalized observation. The normalized log-counts and associated precision weights can then be entered into the limma analysis pipeline, or indeed into any statistical pipeline for microarray data that is precision weight aware[3,4]. Users can check the following publications for more information about methods:
limma : Smyth, G. K. (2004). Linear models and empirical Bayes methods for assessing differential expression in microarray experiments. Statistical Applications in Genetics and Molecular Biology, Vol. 3, No. 1, Article 3.
Voom: Law, CW, Chen, Y, Shi, W, Smyth, GK (2014). Voom: precision weights unlock linear model analysis tools for RNA-seq read counts. Genome Biology15, R29.

4.2 Correlation between gene expression and copy number data

"getCNGECorrelation" function provides correlation coefficient and adjusted p value between copy number and gene expression data for each dataset. This function takes main dataobject as an input (uses gene copy number estimates from GISTIC2 [2] algorithm and gen expression values from every platform (RNAseq and arrays) to prepare return lists. List object stores "CorResult" object that contains results for each comparison.)

```
> #Correlation between gene expression values and copy number
> corrGECN = getCNGECorrelation(dataObject=brcaData,adj.method="BH",
```

```
+                               adj.pval=0.05,raw.pval=0.05)
>
```

If the dataset has RNASeq data, data will be normalized for correlation analysis. Correlation function uses Benjamini Hochberg adjustment for p values. For more details about adjment users can check base adjustment methods by calling "?p.adjust". In addition, to filter results adjusted and raw p values are used. Users can change "adj.pval" and "raw.pval" parameters to refine results. The RTCGAToolbox uses one of Pearson's product moment correlation coefficient to test for associations between paired samples. Measures of association, all in the range [-1, 1] with 0 indicating no association, shows how copy number alterations affect gene expression changes. The test statistic follows a t-distribution, with length (x)-2 degrees of freedom if the samples follow independent normal distributions. Users can get detailed information by calling '?cor.test' function

4.3 Mutation frequencies

"getMutationRate" function gets the data frame that stores mutation frequency for the genes. This function gets the mutation information for each sample that has data and calculates frequency for each gene.

```
> # Mutation frequencies
> mutFrq = getMutationRate(dataObject=brcaData)
> head(mutFrq[order(mutFrq[,2],decreasing=TRUE),])
>
```

4.4 Univariate survival analysis

Survival analysis is considered as one of the methods that can provide clinically valuable information. To provide this information, the function creates 2 or 3 groups based on expression data.(If the dataset has RNASeq data, data will be normalized for survival analysis.). If function is triggered with 2 groups, RTCGAToolbox creates groups using the median expression level of individual genes. If group number is set to be 3, then the groups will be defined as: the samples in 1st. quartile (expression $<$ 1st Q), the samples those have higher expression (expression $>$ 3rd Q) and the samples lying in between these 2 groups.

This function also needs a survival data, which can be obtained using clinical data frame. Clinical data frames can be obtained from main data downloads. First column of the survival data frame should be sample barcodes, second column should be time and the last column should be event data. Below code chunk shows how survival data frame can be obtained from clinical data and how survival analysis can be done.

```
> # Creating survival data frame and running analysis for
> # PIK3CA which is one of the most frequently mutated gene
```

```
> clinicData = brcaData@Clinical
> clinicData = clinicData[3:5,2:ncol(clinicData)]
> clinicData[3,is.na(clinicData[3,])] = clinicData[2,is.na(clinicData[3,])]
> survData <- data.frame(Samples=colnames(clinicData),
+                        Time=as.numeric(clinicData[3,]),
+                        Censor=as.numeric(clinicData[1,]))
> getSurvival(dataObject=brcaData,geneSymbols=c("PIK3CA"),sampleTimeCensor=survData)
>
```

Running the above code will provide following KM plot.

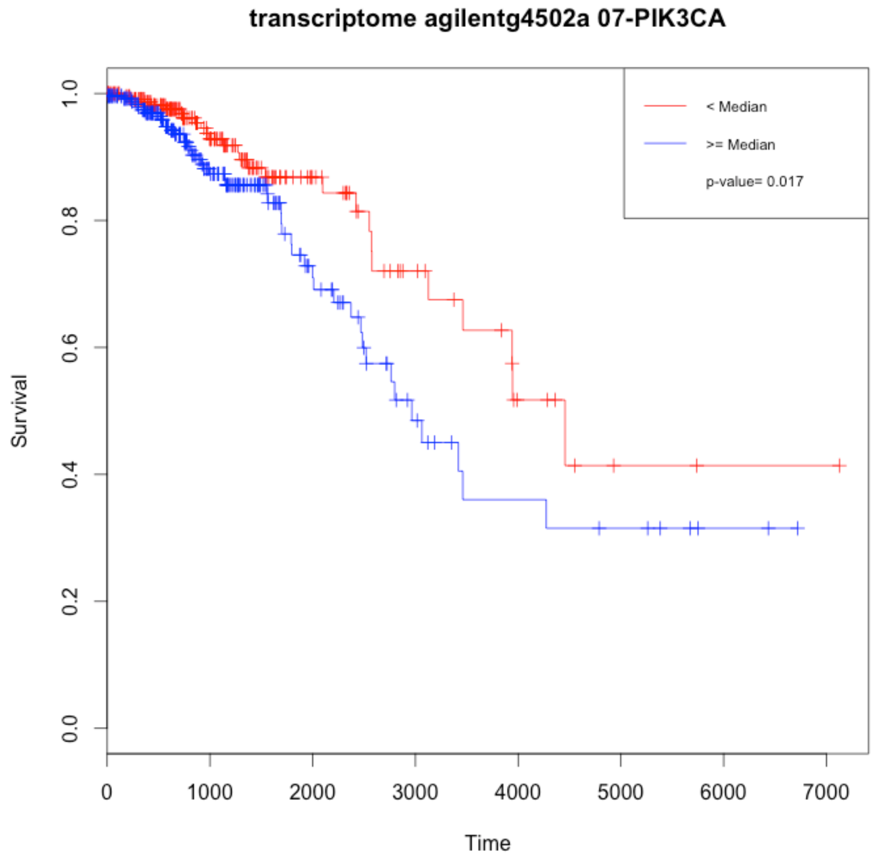

4.5 Report figure

This function provides an overall circle figure for the dataset by using the RCircos[5]. This function uses differential gene expression analysis results (max results for 2 different platforms), copy number data estimates from GISTIC2 [2]

and mutation data.

Outer circle shows the gene symbols that have mutation in at least 5% of the samples. Inner tracks show the significantly altered gene expressions as fold change and copy number changes where blue represents the deletions and red represents the amplifications.

This function needs a genes location data frame, which can be obtained from "hg19.ucsc.gene.locations" data object. Please see the next section.

```
> # Creating dataset analysis summary figure with getReport.
> # Figure will be saved as PDF file.
> data(hg19.ucsc.gene.locations)
> getReport(dataObject=brcaData,DGEResult1=diffGeneExprs[[1]],
+ DGEResult2=diffGeneExprs[[2]],geneLocations=hg19.ucsc.gene.locations)
```

Running code above will provide following KM plot.

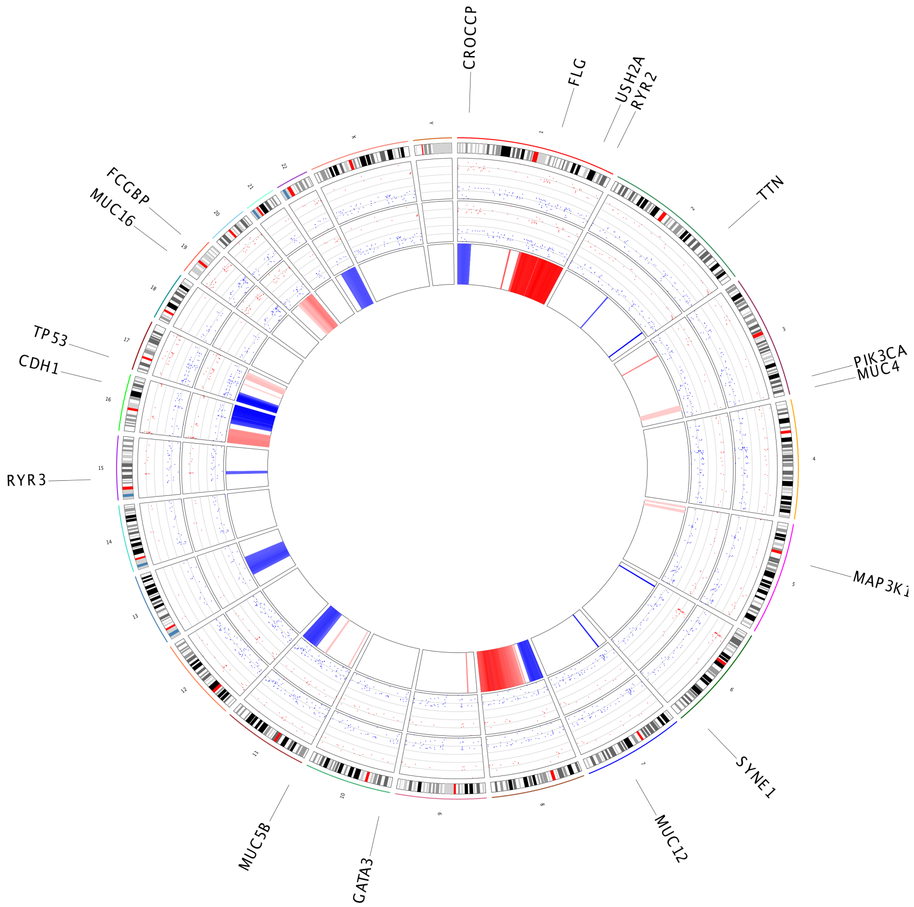

5 Data objects

RTCGAToolbox provides two data objects.

- "RTCGASample" data object provides sample data for testing functions
- "hg19.ucsc.gene.locations" data object provides required gene coordinates from hg19 for report figure.

```
> data(RTCGASample)
> data(hg19.ucsc.gene.locations)
```

6 sessionInfo

```
> sessionInfo()

R version 3.0.2 (2013-09-25)
Platform: x86_64-apple-darwin10.8.0 (64-bit)

locale:
[1] en_US.UTF-8/en_US.UTF-8/en_US.UTF-8/C/en_US.UTF-8/en_US.UTF-8

attached base packages:
[1] stats     graphics  grDevices utils     datasets  methods   base

other attached packages:
[1] RTCGAToolbox_0.99.0

loaded via a namespace (and not attached):
[1] limma_3.18.13   RCircos_1.1.2   splines_3.0.2   survival_2.37-4
[5] tools_3.0.2     XML_3.95-0.2
```

References

[1] Cancer Genome Atlas Research N (2008) Comprehensive genomic characterization defines human glioblastoma genes and core pathways. Nature 455: 1061-1068.

[2] Mermel CH, Schumacher SE, Hill B, Meyerson ML, Beroukhim R, et al. (2011) GISTIC2.0 facilitates sensitive and confident localization of the targets of focal somatic copy-number alteration in human cancers. Genome Biol 12: R41.

[3] Smyth GK (2004) Linear models and empirical bayes methods for assessing differential expression in microarray experiments. Stat Appl Genet Mol Biol 3: Article3.

[4] Law CW, Chen Y, Shi W, Smyth GK (2014) Voom: precision weights unlock linear model analysis tools for RNA-seq read counts. Genome Biol 15: R29.

[5] Zhang H, Meltzer P, Davis S (2013) RCircos: an R package for Circos 2D track plots. BMC Bioinformatics 14: 244.
